# Estimating population-based coverage of reproductive, maternal, newborn, and child health (RMNCH) interventions from health management information systems: a comprehensive review

**DOI:** 10.1186/s12913-021-06995-z

**Published:** 2021-10-25

**Authors:** George Mwinnyaa, Elizabeth Hazel, Abdoulaye Maïga, Agbessi Amouzou

**Affiliations:** Department of International Health, Johns Hopkins Bloomberg School of Public Health, 615 N. Wolfe Street, MD 21205 Baltimore, USA

**Keywords:** RMNCH, Coverage, HMIS, LMICs, Review, Reproductive, Maternal, Neonatal, Child, Health

## Abstract

**Background:**

Routinely collected health facility data usually captured and stored in Health Management Information Systems (HMIS) are potential sources of data for frequent and local disaggregated estimation of the coverage of reproductive, maternal, newborn, and child health interventions (RMNCH), but have been under-utilized due to concerns over data quality. We reviewed methods for estimation of national or subnational coverage of RMNCH interventions using HMIS data exclusively or in conjunction with survey data from low- and middle-income countries (LMICs).

**Methods:**

We conducted a comprehensive review of studies indexed in PubMed and Scopus to identify potential papers based on predefined search terms. Two reviewers screened the papers using defined inclusion and exclusion criteria. Following sequences of title, abstract and full paper reviews, we retained 18 relevant papers.

**Results:**

12 papers used only HMIS data and 6 used both HMIS and survey data. There is enormous lack of standards in the existing methods for estimating RMNCH intervention coverage; all appearing to be highly author dependent. The denominators for coverage measures were estimated using census, non-census and combined projection-based methods. No satisfactory methods were found for treatment-based coverage indicators for which the estimation of target population requires the population prevalence of underlying conditions. The estimates of numerators for the coverage measures were obtained from the count of users or visits and in some cases correction for completeness of reporting in the HMIS following an assessment of data quality.

**Conclusions:**

Standard methods for correcting numerators from HMIS data for accurate estimation of coverage of RMNCH interventions are needed to expand the use of these data. More research and investments are required to improve denominators for health facility-derived statistics. Improvement in routine data quality and analytical methods would allow for timely estimation of RMNCH intervention coverage at the national and subnational levels.

## Background

Many countries have made significant progress in increasing the coverage and quality of maternal, newborn and child health services during the era of Millennium Development Goals (MDGs) and this is also true for countries that did not achieve the MDG 4 and 5 [1, 2]. As low- and middle-income countries (LMICs) strive to maintain the gains and progress towards achieving the Sustainable Development Goal 3 (SDG 3), there is a need to use rigorous analytical methods to analyze readily available routine health facility data to track coverage of key health indicators both at the national and subnational levels.

The coverage of a reproductive, maternal, newborn, and child health (RMNCH) intervention is defined as the proportion of the population in need of the intervention or service that actually receives it[3]. The percentage of the target population that received the defined RMNCH intervention (RMNCH intervention coverage) is a crucial measure in public health[4]. At the global and country level, RMNCH intervention coverage indicators are used to monitor progress, identify coverage gaps, allocate resources, plan future interventions and guide health policies[5]. Additionally, RMNCH intervention coverage indicators are also used to determine countries eligibility for global support programs such as performance-based financing[6–8], support from the GAVI Alliance for the introduction of new vaccines [9], the Millennium Challenge Account assistance[10] and other international support programs[11].

The number of individuals who received the RMNCH intervention (numerator) can be used to examine trends over time and difference between subnational units, but the estimation of coverage requires a denominator. In most LMICs, RMNCH intervention coverage based on health management information systems (HMIS) data is estimated using aggregate routine health facility reports and the estimated target population from census projections[4]. When a person visits a health facility for routine RMNCH care, the health worker records the service provided in their clinic register (facility-held record), a tally sheet and often also on a home-based record that the individual keeps with them[12]. Health workers at each health facility compile data manually at the end of each month from the facility registers and tally sheets, using standardized reporting forms which are forwarded to their subnational unit (such as district) health department. The latter office enters the data into the computer and a web-based system (HMIS) such as DHIS2 which is used to check data quality, produce scorecards, and transfer data to regional and national Ministry of Health.

The recording, transcription, compilation and reporting of RMNCH data from health facilities is highly prone to errors and misreporting[12–18]. Reports based on health facility data may underestimate population level intervention coverage due to incomplete reporting from reporting facilities or non-reporting from other RMNCH intervention providers (e.g. private sector, non-governmental organizations)[12, 19]. Similarly, reports may overestimate coverage due to overreporting from facilities (due to inclusion of people outside the target group for the intervention) or movement of people across administrative boundaries (districts, regions)[19, 20]. The further use of RMNCH intervention coverage to determine eligibility for international support also promotes inflation of administrative coverage estimates[21]. For some RMNCH interventions such as immunization, doses distributed (not the actual number of people receiving the intervention) are sometimes used as the numerator which does not take wastage into account[20, 22–24].

In addition to numerator challenges, denominators derived from census and civil registration and vital statistics (CRVS) are often grossly inaccurate[14]. The availability of census data from multiple time points together with accurate CRVS data is necessary for accurate population projections. However, the availability of these data are limited particularly in Sub-Saharan Africa and Asia. Globally, it is estimated that only 59 % of infants younger than 12 months have their births registered and only 33 % in Sub-Saharan Africa and Asia[25]. Globally, 53 % of deaths are not registered and the proportion of unregistered deaths is likely to be much higher in LMICs[25]. Additionally, there is limited information on migration in LMICs making it impossible to adjust for that in estimating target population for the various RMNCH interventions.

The availability of recent and appropriate population level census data is rare in many LMICs[26]. For example, the Democratic Republic of Congo[27], Ethiopia[28], Burkina Faso[29], and Afghanistan[30] held their latest population census in 1984, 2007, 2006 and 1979, respectively. In other countries like Nigeria, not only is the census data old, but the accuracy is highly contested[31, 32]. Given these challenges, the population projection for key RMNCH indicators using census data in LMICs is problematic. When the denominator for a defined intervention is overestimated or under-estimated, the resulting coverage becomes unexpectedly low or high. It is common to see estimated RMNCH indicator coverage from LMICs exceeding 100 %, partly as a result of incorrectly estimated target population (denominator too low) or numerator (too high)[5, 26, 33].

Given the limitations on coverage estimates derived from health facility data, household surveys are considered the ‘gold standard’ for the measurement of RMNCH intervention coverage and are also used to validate coverage estimations from health facility data[3]. However, household surveys have their own biases and limitations[20]. They are infrequent, expensive, time and resource intensive, and are generally not powered for finer subnational coverage estimates limiting their usefulness[3, 5, 20]. They are limited in measuring some conditions (e.g. pneumonia in children) and their treatment coverage, and are also affected by inaccuracies in the measurement of several maternal and newborn health coverage indicators[34–37].

Policy makers and public health officials across LMICs rely on RMNCH intervention coverage estimates from routine data to timely identify coverage gaps. These data are also used to ascertain the effects of unexpected events on service use such as the current Covid-19 pandemic. It is becoming increasingly necessary to identify reliable analytical methods for measuring the coverage of RMNCH interventions using HMIS data. Inaccurate estimation of the numerator and/or denominator will result in wrong coverage estimates, misinform national and local health policies and result in wrongful allocation of scarce resources. There is limited information on the most up-to-date available analytical techniques that are used to address the limitations of HMIS data and to improve the estimate of RMNCH intervention coverage using HMIS data.

In this paper, we carried out a comprehensive review of the literature to identify approaches that have been used for estimation of national or subnational coverage of RMNCH interventions using HMIS data exclusively or in conjunction with survey data in LMICs. We describe these methods and highlight their strengths and limitations. This information will be useful to researchers, ministries of health and local health departments in LMICs who continuously strive to improve the accuracy of RMNCH intervention coverage estimates using their local HMIS data. To the best of our knowledge, this is the first comprehensive review focusing on analytical methods to improve estimation of RMNCH intervention coverage using routine data.

## Methods

We focused on methods used to assess internal and external consistency of HMIS data for the estimation of RMNCH intervention coverage, together with ways of handling incomplete reporting, estimating the target population, and handling of data inconsistencies. We classified denominator estimation methods into two groups: census-based projections and non-census-based projections. Census-based projection is the estimation of a target population using population-based projections (e.g. population censuses, CRVS) with or without further adjustments. Non-census-based projections include the use of data from other sources besides census-based projections such as survey data, reports from other indicators or from previous coverage information.

### Search strategy

To reduce the potential of missing relevant articles, broad search terms were used. We searched PubMed and Scopus using several search terms (Table 1). Titles and abstracts containing terms such coverage, routine health data or district health management information, national, or subnational that are indexed in PubMed or Scopus were included in the search. All relevant articles published in any language (with titles or abstracts translated in English) prior to September 2019 were reviewed.

**Table 1:** Search Strategy

Databases Searched: PubMed and Scopus	
Keywords	region, national, subnational, nationwide, nation-wide, sub-national, district, state, states, statewide, province, provincial, province-wide, provincewide, local, city, citywide, prevalence, prevalence, scale up, at scale, coverage, penetration, extent, health information systems, health management information system, routine health data, routine health information, district health information software, health-facility data, facility data, routine data, administrative data, program data, HMIS, DHIS, RHIS

### Inclusion and exclusion criteria, and review procedure

Original articles were included in the review if they estimated coverage of any RMNCH related indicator such as immunization, antenatal, stillbirth, delivery and postnatal services, family planning services, management of childhood illness, prevention of mother-to-child transmission of HIV, and using routine data alone or together with any survey data. Letters to the editor, editorials, reviews or systematic reviews, meta-analysis, case reports and articles that used administratively reported coverage without indicating how the numerator or the denominator was estimated for the reported coverage were excluded.

The title and abstract of each article were first reviewed, followed by a full text review. Each article was reviewed by two independent reviewers and when discrepancies occurred, the reviewers met to discuss and resolve all disagreements. Covidence software was used to manage the review of the articles[38].

### Data extraction

The following information was extracted from each of the articles included in the review: year of publication, indicator for which coverage was estimated, location of study, whether the estimated coverage was at the national or subnational level, whether the coverage estimate was exclusively from routine data or used a combination of information from routine and survey data, estimation of target population (denominator) and numerator. Other key features identified were documented as well. We focused on the methods to adjust for the denominators and numerators, missing data, over and under reporting and the overall coverage estimate. In cases where the study had multiple indicators, only the methods applied to the estimate of RMNCH related indicator coverage were considered.

The coverage estimation methods were divided into two groups based on whether the coverage was for preventive or curative indicator as these typically require different approaches for denominator estimation. Curative-based coverage refers to indicators for which the denominator refers to a target population with a specific disease or condition presented at the clinic. These include, for example, antiretroviral treatment for HIV positive pregnant women for the purpose of preventing mother-to-child transmission or treatment of illnesses in children under five years of age. Preventive-based intervention coverage refers to coverage for interventions that are not related to treatment of an active disease or condition; examples include immunization of children, family planning services, vitamin A distribution, and antenatal and postnatal services.

## Results

**Fig. 1:**
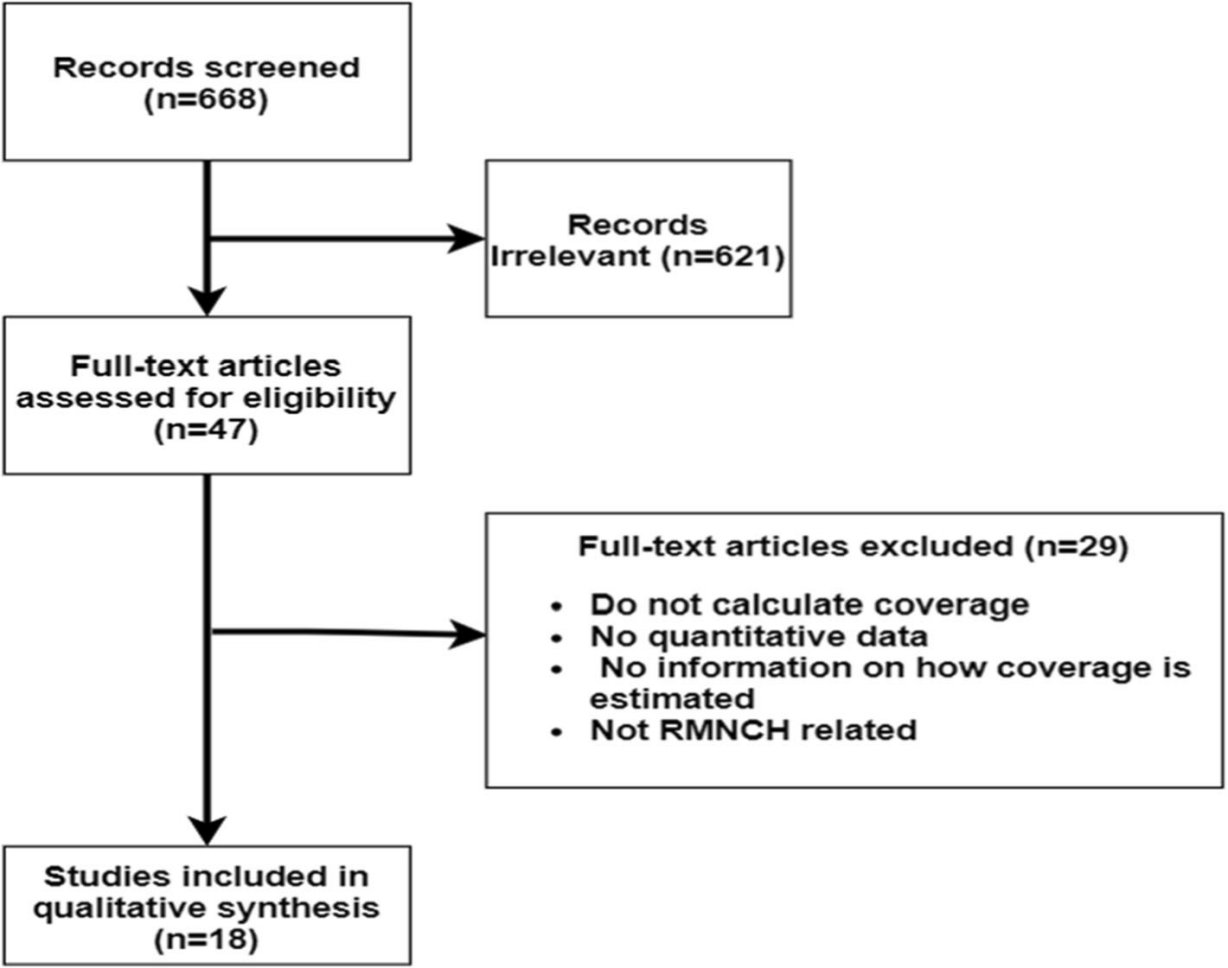
Flow chart showing the screening process

Figure 1 shows the screening process. The initial search identified 668 articles. Of the 668 articles 621 (93 %) were excluded after title and abstract review because they were not related to RMNCH or coverage estimation. Full review of the remaining 47 articles resulted in the exclusion of 29 (62 %) articles because they did not calculate coverage or they used reported coverage from other sources without providing any additional information on how the numerator and the denominator of the coverage were estimated. Of the 18 articles included in this review, 12 (67 %) used only routine data and 6 (33 %) used both routine and survey data to estimate coverage for RMNCH indicators. Table 2 provides a summary of all studies included in the review.

**Table 2:** Characteristics of papers identified during the literature search

Reference (year) country	Indicator(s)	Denominator	Numerator	Coverage estimate	Data quality approach
Agyepong et al. (1999) Ghana	BCG, Measles, DTP3 & OPV3 vaccination	From health department	Reports from district health department	Took the coverage reported by the local health department	No reports on data quality assessment or adjustment
Assegaai et al. (2018) South Africa	ANC attendance, Measles vaccination, vitamin A, contraceptive coverage	Census projections (Population aged < 1 year, 1–5 years & 15–49 years, respectively	Count of individuals who received the service	Divided adjusted numerator by denominator	Adjusted for missing reports and outliers
Audureau et al. (2013) 15 African countries)	Anti-retro viral drugs for PMTCT	Number of women attending ANC multiplied by HIV seroprevalence at each site	Number of women receiving ART at each site	Divided numerator by denominator	No reports on data quality checks or adjustment
Bhatnagar et al. (2016) India	DTP3 vaccination	Used a combination of subnational routine coverage reports together with available coverage estimates from surveys to estimate national level immunization coverage		Sum of weighted state immunization coverage	The goal was to estimate national level immunization coverage using the WUENIC method, assessed and made several assumptions on missing data and outliers
Borgdorff and Walker (1988) Zimbabwe	Measles vaccination & ANC attendance	Census projections: crude birth rate, number of children under 12 months of age	Number of immunizations administered, number of ANC attendance	Divided numerators by denominators	No reports on data quality checks, missing reports or adjustments
Delvaux et al. (2011) Cambodia	HIV & Syphilis testing among pregnant women, ANC, facility deliveries, family planning	Census projection: crude birth rate, women of reproductive age	Number of pregnant women tested for HIV & Syphilis, number of pregnant women attending at least one ANC visit	Divided numerator by denominator	Reported checking for data quality but no reports on handling quality challenges, missingness, outliers, etc.
Dunkle et al. (2014) Nigeria	DTP3 vaccination	Census projections: reported number of live births	Doses of DTP3 and Measles administered	Divided numerator by denominator	Assessed for incomplete reporting, outliers, inconsistencies, but did not do anything to address the identified challenges in the analysis
Haddad et al. (2010) Burkina Faso	DTP3 & Measles vaccination	Census projection	Number of children 0–11 who received the antigen	Divided numerator by denominator	Discussed the limitations and data quality issues but did not do anything to address the identified challenges in the analysis
Jeffery et al. (2018) Madagasca & Benin	Polio vaccination & Vitamin A	Census projection: coverage from previous campaign	Number of children (6–11 months and 12–59 months) who received Polio vaccine and Vitamin A supplement, respectively	Combined coverage from routine and survey data using a hybrid estimator	The hybrid estimator is a weighted average of the reported coverage from routine and survey data, the hybrid estimator produces standard errors and 95 % confidence intervals
Lacapere et al. (2011) Haiti	Measles, Rubella, Polio vaccination & Vitamin A	Census projections	Number of people who received the vaccine or supplement	Divided numerator by denominator	Assessed for data quality during implementation of the campaign not at the analytical stage
Maina et al. (2017) Kenya	Pentavalent vaccine, ANC visit, health facility delivery, C-section	Census projections, surveys, coverage of related indicators	Reported number of people who received the intervention of interest	Divided adjusted numerators by the different denominators	Did extensive data quality assessment and adjusted for the various quality issues
Mensah et al. (2019) Madagascar	Measles vaccination	Census projections	Number of doses administered	Divided numerator by denominator	No reports on data quality assessment or adjustment
Nanyunja et al. (2003) Uganda	Measles vaccination	Census projections	Number of doses administered	Divided numerator by denominator	Assessed for incomplete reporting but did not adjust that in coverage estimate
Saito et al. (2018) 7 East and Southern African countries	Pediatric HIV treatment	Estimated number of children living with HIV (from survey)	Number of HIV positive children on ART (from routine data)	Divided numerator by denominator	Assessed and adjusted for missing routine data
Vivancos & Martinez (2008) Uganda	DTP1 & DTP3 vaccination	Census projections	Number of doses administered	Divided numerator by denominator	Did not report data quality assessment or adjustment
Wandera et al. (2018) Kenya	Rotavirus vaccination	Census projections	Number of children immunized	Divided numerator by denominator	Did not report data quality assessment or adjustment
Wandera et al. (2017) Kenya	Rotavirus vaccination	Census projections	Number of children immunized	Divided numerator by denominator	Did not report data quality assessment or adjustment
Zuber et al. (2003) Burkina Faso		Census projections: used coverage from previous campaigns and adjust using census information	Number of doses administered	Divided numerator by denominator	Did not report data quality assessment or adjustment

DTP: diptheria, Tetanus, pertussis; ANC: Antenatal care; HIV: Human immuno-deficiency virus; OPV: oral polio vaccine; BCG: Bacillus Calmette-Guerin; PMTCT: prevention of mother to child transmission

### Estimation of denominators

Seven studies took the denominator as reported by the subnational health administration or the national institute of statistics without providing details on how it was estimated[5, 22, 39–43], 8 studies used the census based estimation methods[23, 24, 33, 44–48], 4 used non-census-based estimation approach[5, 24, 49, 50] and 3 used a combination of census and non-census-based estimation approach depending on the indicator and the available data[24, 51]. Denominator estimation for curative-based interventions were limited to only non-census-based methods[49, 50].

Census-based projections: Census based parameters including population growth rate, crude birth rate and mortality rate were used independently or together to estimate the expected population in need of a defined RMNCH service (denominator) at the subnational level. In census based denominator projections, population based parameters such as growth rate and fertility rate are applied to the most up to data population census figures derived from a single time point to project the size of the population at subsequent time points. Census-based denominator estimation methods were mostly limited to preventive services for which the target population can be generated from population census data. These include, for example, births for ANC visits, facility deliveries or surviving infants for immunization rates. Studies that used population growth rate or crude birth rate to estimate the target population did not indicate whether such rates were national or subnational parameters and did not indicate how the growth rate was estimated[23, 47, 50].

Depending on the RMNCH intervention of interest, several studies used different census-based parameters and approaches to estimate the target population. As described in Table 1 the population growth rate is applied to the population reported from the most recent census to obtain the projected population of the time of interest. The reported proportion of the target population from the most recent census is multiplied by the projected population to determine the target population for the time of interest[24, 44–46].

Instead of applying the growth rate to the total population, some authors applied the growth rate to the target population reported from the most recent census (proportion of the total population that falls within the target population). This approach was used by Haddad et al. to estimate the number of children less than 12 months of age for the purpose of estimating DPT3 and measles coverage for year 2002/2003 across multiple districts in Burkina Faso. The authors used the national population growth rate estimated from the 1996 census to project the population of children less than 12 months of age in each district, thus ignoring between district variations in both the growth rate and the target population. The population growth rate and the proportion of the target population were considered constant from when the census was done and this was true for other papers[23, 46, 48]. Dunkle et al. used a similar approach to estimate the coverage of DPT3 and measles in Nigeria but varied the proportion of surviving infants each year using state specific infant mortality (or proportion of surviving infants) data as reported by the state health department[33].

For reproductive health indicators (coverage of ANC attendance, antenatal HIV and syphilis testing, facility deliveries), Delvaux et al. multiplied the estimated crude birth rate from survey data by the census projected population of the subnational unit of interest to determine the number of expected pregnancies[48].

Non-census-based estimation methods: Non-census-based denominator estimation approaches relied on other sources using different techniques to estimate the population in need of the service. Combined and non-census-based denominator estimation methods are described in Table 3 below. Denominators were derived using related RMNCH interventions (e.g. DTP1 and DTP3), reported health facility users, previous coverage information, and combining previous coverage with census projections. This approach was mostly used to estimate denominators at the subnational level.

**Table 3:** Combined and non-census-based denominator estimation methods

Approach	Description and application	Limitations
**Population census-based denominator estimation methods**		
Census-based projections	First, the population growth rate of the defined location obtained from the most recent census report or the subnational unit statistical office was applied to the population reported from the most recent census to obtain projected population for the time period of interest. The proportion of the total population that falls within the target population as reported from the most recent census is applied to the projected population to determine the population in need of the defined RMNCH intervention[24, 44–46]. This method was used by several authors in different ways depending on the RMNCH indicator of interest and mostly limited to preventive services.	Census may be too old, and projections may not be accurate particularly in LMICs. Using national level growth rate for subnational estimates and treating growth rate as constant overtime is problematic.
**Non-census-based denominator estimation methods**		
Using other RMNCH reported data to estimate the target population for the indicator of interest	This approach first identifies service indicators with similar target population for which existing household surveys show near universal (100 %) coverage level. The target population is then obtained from the facility data and adjusted slightly for the level of non-facility use and mortality in the population[5]. This method was used by Maina et al. and applies mostly to preventive coverage indicators[5]. To estimate the denominator for immunizations given at birth, reported ANC1 attendance can be used to estimate the number of births expected and vise verse[41]. Similarly, to estimate the coverage for DPT3 or OPV3, the target population can be estimated from the reported number of children who received DPT1, OPV1 or BCG within the same time period[5, 41].	Requires high coverage of related indicators; using regional level coverage to estimate denominators at the district level does not account for variation across districts.
Facility user-based denominators	This method is a multistep approach that is used to estimate the denominator for clinical interventions. First, a defined group of people accessing regular preventive care are first tested to determine if they have the condition of interest (prevalence of the condition among a subset of all who are seeking regular preventive service at a facility). The estimated prevalence of the condition among the subset tested is then applied to the total number of people accessing the defined preventive care to determine the total population of people in need of service for the tested condition. This method was used by Audureau et al. to estimate the coverage of antiretroviral drug coverage among HIV positive pregnant women for the purpose of preventing mother-to-child transmission. For instance, to estimate the denominator for the coverage of a single dose of nevirapine among HIV positive pregnant women, the authors multiplied the number of women attending ANC at each site by the observed HIV seroprevalence at each site (number of HIV-positive pregnant women/number of HIV tested pregnant women)[49].	Under-estimate pop in need by ignoring non facility users; unlikely appropriate for Low income countries where facility access and use is low
Using survey data	Like the facility user-based estimates, this approach applies survey reported prevalence of the condition of interest to the projected total population at the subnational level to estimate the population in need of service for the condition of interest. Saito et al. used this approach to estimate anti-retroviral therapy (ART) coverage for HIV infected children[50].	Requires frequent and accurate census data which is a challenge in LMICs; using regional level coverage from survey to estimate denominators at the district level does not account for variation across districts
Using previous coverage information	This approach is used for shorter campaign-based health interventions where the denominator is unknown but there is information from previous campaigns on the same intervention. The authors did not provide any additional explanation on how this was done[47].	Does not account for changes in the target population from when the last campaign was conducted.
**Combined census and non-census-based approaches**		
Using previous coverage and census projections	Zuber et al. used this method to estimate the target population for a polio campaign program. First, the authors retrieved the number of people who received the intervention during the previous campaign, then multiplied the number of people who received the intervention during the last campaign by a multiplicative factor corresponding to the average annual population increase of the geographic location of interest. The authors did not provide details on how the annual population growth was estimated and whether the previous coverage was considered to be 100 %[24].	Requires data from previous campaign; assumes that coverage from previous campaign is 100 %; Using national level growth rate for subnational estimates may not be appropriate; no details on the estimation process
Census projections, previous coverage estimates, infant mortality and expert panel	This approach combines census projections with previous coverage data, infant mortality, and expert panel to estimate the denominator. First, the population in need is estimated using the population growth rate from the most recent census (as described earlier). Expert panel consisting of health administrators will then use previous coverage information and the number of reported deaths to adjust the census projected target population. This method was used by Mensah et al. to estimate measles coverage in Madagascar [51]. The authors did not provide details on how the previous coverage or infant mortality is used in this process.	In addition to the census based and previous campaign data limitations outlined above; the use of input from expert panel may result in arbitrary adjustment which may vary from year to year making it impossible to look at trends overtime or performance between subnational units

### Estimation of numerators

The methods used for the estimation of numerators were very similar across indicators. For most of the RMNCH indicators, the numerator was simply taken from the health facility, district or province as reported number of people who received the service with or without any further quality checks or adjustments. This was done mainly for interventions that are largely delivered at the facility level or for which service statistics are well compiled. Numerators for the same indicators were defined differently by study authors. For immunization coverage, for example, some authors used the counted number of children immunized with the specific antigen of interest as the numerator[39, 40, 44] and others used the number of doses distributed to estimate the numerator[22–24]. Some studies did not assess for quality of the reported numerator and for those that did, nothing was done about the identified problems[33, 42, 45, 46]. However, a few studies used different techniques to assess and address the identified quality issues[5, 48].

### Assessing and addressing data quality issues in numerators

Table 4 provides details on some of the approaches used at the analytical stage to address data quality challenges. Several studies acknowledged the challenges with using routine data to estimate coverage but did not provide any suggestions on how to handle such challenges at the analytical stage. However, few studies (5/18) used the World Health Organization (WHO) recommended facility data quality review tool[52] together with additional methods to assess and adjust for challenges such as missing/incomplete data, extremely high or low coverage estimates (outliers) and inconsistent reporting. These challenges were addressed differently when estimating coverage at the national and subnational level. Incomplete reporting happens when a facility fails to submit their monthly report to the health administration for a given period. However, incomplete reporting can also occur when a facility provides RMNCH services but is not required or does not send reports to the sub-district health office[5]. Two methods were used to assess for internal consistency of the reported indicator: examining trends of the reported numerator over time or comparing the reported numerator between indicators that are expected to be similar (e.g. ANC1 and DPT1) or monotonic ( e.g. DPT1 vs. DPT3)[5, 41]. There were two main approaches to handling outliers and missing reports.

The first method is to make assumptions about the incomplete/missing data and then adjust for the reported data based on the assumption. The second approach is to define a threshold for missingness and apply different rules for missingness above or below the set threshold. If missingness for a given facility is greater than the threshold, they are removed from the analysis. If missingness is less than the threshold, replace missing reports with an average of the months where data was reported. For outliers, first define an outlier with respect to the reported data (any reported data that is 2 or 3 standard deviations from the mean of all reported data for the period). Then state how outliers will be handled; for example, replace with mean reported value, remove from analysis, etc.

**Table 4:** Addressing data quality challenges

Data Quality Issue	Approach	Limitations
Missing/incomplete reporting	Maina et al. proposed a method to use an adjustment factor known as the k-factor to correct for incomplete reporting by making an assumption on the number of people served by the facilities that did not report compared to the number of people served by facilities that reported the defined intervention to the local health administration. The k-factor is heavily influenced by the extent to which large health facilities as well as private sector facilities are reporting and engaged in the provision of service for the intervention of interest in the given location[5]. This method was used to address numerator challenges for preventive interventions.	The k-factor is often arbitrarily determined from HMIS officers and may not reflect the truth
Missing monthly report from a reporting facility	Assegaai et al. used defined rules to decide how to handle such data depending on the level of missingness [41]. If a facility missed more than 8 monthly reports for a given year, they were removed from estimation of coverage at the subnational level. However, if a facility missed less than 9 monthly reports, the average value of the months where data were reported was assigned to the months with missing data. The authors did not indicate how they arrived at the defined cut offs or provide any justification for the selected actions on missing data[41]. This method was used to address numerator challenges for preventive interventions.	Threshold for missingness and decisions on how to handle missingness is subjective making it difficult to compare estimates across different subnational units with different missingness threshold and decisions on how to handle such missingness.
Inconsistent reported numerators	Maina et al. defined outliers as reported numerators that are more than 2 standard deviations of the mean reported numerator for the multi-year period and such outliers were adjusted if there were no reasonable justifications. The authors did not state how the adjustment was done [5]. The authors did not indicate what was considered as a “reasonable justification” for outliers. Assegaai et al. handled inconsistencies in reported numerators differently. For district or county level RMNCH coverage estimation, if the reported value from a facility in a given month was extremely higher (> 3 times) or lower than the yearly average numerator reported for the given indicator by the facility, the outlier was replaced with the average yearly reported value of the selected indicator from the given facility. This methods were used to address numerator challenges for preventive interventions.	The definition of outliers and how to handle such data varies making it difficult to compare coverage overtime and between subnational units

### Estimation of subnational and national RMNCH intervention coverage

In many instances (14/18) the numerator reported by the facility was divided by the estimated denominator (mostly from census projections) to calculate the coverage of the indicator of interest without any further adjustment. However, other authors used a combination of techniques and data sources to estimate coverage for RMNCH indicators. The methods for estimating coverage at the subnational level were slightly different from the methods used for national level coverage estimates. Surveys such as the Demographic and Health Survey and the Multiple Indicator Cluster Survey are powered to estimate coverage at both national and the first administrative level (region/province). As a result, national RMNCH intervention coverage estimation combined routine coverage estimates from the subnational level with survey estimates to produce a national estimate. Subnational level coverage estimates depend mainly on routine facility data and census projections with limited options or availability of finer subnational (district level) survey data.

Jeffery et al. proposed the annealing technique which uses a hybrid estimator, a weighted average of the estimated coverage from administrative and survey data. The hybrid estimator produces standard errors and 95 % confidence bounds around the coverage estimate[47]. Maina et al. used the adjusted numerator and denominators from multiple sources described above together with several assumptions (census projected denominators, denominators from indicators with high coverage and survey projected denominators) to estimate different coverage for each intervention and to compare the estimated coverage across estimated denominators to determine the most plausible coverage estimate[5].

Bhatnagar et al. used an approach similar to the World Health Organization/UNICEF (WHO/UNICEF) Estimates of National Immunization Coverage (WUENIC) approach to estimate national level coverage of immunization. The WUENIC method uses data from multiple sources including administratively reported data, survey data and expert panel input to determine a national estimated coverage for immunization. In this approach, the administrative coverage data from the subnational level are used in the national estimate if there is no coverage estimate from other sources or coverage data that differed by 10 % points from the subnational administratively estimated coverage. Data from any subnational unit (state) that reported over 100 % coverage or had high year-to-year variations in coverage (unexpectedly low or high coverage) were considered inaccurate unless there was a justification from the state as to why there were such variations (vaccine stock out, health workers on strike, etc.). The authors made several other assumptions on the different data quality challenges[43]. To estimate the national immunization coverage, a subnational weighted coverage estimate was calculated by weighting the subnational level coverage with population weights defined as the number of reported births from each subnational unit divided by the total number of reported births in the country.

## Discussion

The purpose of this review was to identify the methodologies that have been used for estimation of national or subnational coverage of RMNCH indicators using HMIS data exclusively or in conjunction with survey data from LMICs in order to inform local public health officials on available coverage estimation options. In this review, the methods used to estimate the population in need (target population) and the population who received the intervention (numerator) were different even when the geographic scope and the indicator for which coverage was estimated were the same. Similarly, methods to assess data quality, adjust for incomplete/missing data and handling of data inconsistencies also varied across studies. Almost all reviewed articles acknowledged the challenges of using routine data to estimate RMNCH indicator coverage, particularly at the subnational level. The findings from this review demonstrate that while there may not be a perfect ‘gold standard’ method available to fix the quality issues associated with routine data at the analytical stage, there are existing tools that can be applied to improve the quality of administrative data to ensure that coverage estimates from such data accurately represent the true coverage at the population level.

The current WHO guidelines provide tools on the definition of indicators and how to assess data quality including inconsistencies, missing/incomplete reporting, over/under reporting and how to easily identify errors, especially for reported numerators[53, 54]. However, there are limited guidelines and standard on how to handle such challenges at the analytical stage. This lack of standards leads to varying and subjective assumptions resulting in different coverage estimates. Depending on the challenge, some authors excluded troubling data from the analysis or applied subjective assumptions that may not be realistic and reproducible. For instance, some authors excluded all facilities missing more than 8 monthly reports and made several assumptions on facilities missing less than 9 monthly reports. Additionally, some authors defined outliers as 2 standard deviations while others defined outliers as 3 standard deviations from the mean reported data for the reporting period. Using expert panel input and making assumptions and definitions on outliers limit the ability to appropriately compare immunization coverage across subnational units or trends over time since different year to year assumptions will result in different coverage estimates. Similarly, when subnational units make different assumptions or handle data quality issues differently, the resulting coverage estimate may not be comparable across subnational units. There is the need to standardize how to handle the challenges with routine data at the analytical stage which will allow for appropriate trend analysis and comparison between subnational units.

Notable challenges remain in the definition and estimation of RMNCH indicator numerators. Some authors used the reported dose as the numerator to estimate immunization coverage. Using the number of doses administered does not account for vaccine wastage and may result in over-estimation of the numerator.[3] With indicators like ANC attendance, family planning services, and antiretroviral treatment for HIV positive pregnant women the reported numerator from the facility level is used without any modification[48]. In situations where such services are provided by other non-reporting sources such as drug stores or private entities, using the reported numerators from government facilities could result in gross underestimation of the intervention coverage in the population.

A troubling assumption made by some authors is the idea that any reported RMNCH indicator coverage that exceeds 100 % is a sign of poor data quality. Indeed, some authors excluded all reported subnational coverage that exceeded 100 % (without explanation) from the estimation of RMNCH indicator coverage at the national level. It is acknowledged that routine data may be inflated particularly when high coverage attracts rewards or incentives[20, 21], however, not all high coverage may indicate poor data quality or inflated data. Internal migration (due to conflict, hunger, etc.), providing service to people outside the recommended target population, and mismatch between the distribution of health facilities and administratively defined geographic boundaries (districts, regions) can result in estimated RMNCH intervention coverage exceeding 100 %[19, 24]. In several LMICs, children or pregnant women can receive services such as immunizations from any part of the country. For instance, when a pregnant woman or a child receives immunization or other RMNCH intervention from a district/region outside their area of residence, they are counted in the numerator but not in the denominator of the reporting subnational unit. Health facilities and subnational units do not report or capture the number of people receiving RMNCH related service who are coming from outside the reporting subnational unit. Not reporting or accounting for such individuals would not be a problem if the movement across administrative boundaries was even, which is unlikely. In an ideal situation where the target denominator is correct, and a subnational unit is able to deliver the specific RMNCH intervention to all its target population plus individuals not accounted for in their denominator (people from other administrative areas), the resulting coverage will correctly exceed 100 %. This scenario is applicable to all RMNCH interventions.

Hence, there is the need for future studies to examine the movement of people across subnational units and to determine if the number of individuals accessing RMNCH related services out of a defined area is equivalent to the number coming into the specific subnational unit from other catchment areas to access the same service. This will make it possible to account for the imbalanced movement of people across catchment boundaries and will also help to delineate genuine high coverage from over-reporting or poor data quality related coverage estimates.

There has been some advancement in improving the estimation of denominators for RMNCH indicators using direct census projections or indirect estimation methods. Direct census-based projections will provide a reliable estimate of the target population if the census data is recent, segregated and the projections are done correctly. However, if the population census is old, which is common in LMICs, the projections can be unreliable, particularly at the subnational level, resulting in over- or under-estimation of the target population[5]. Treating growth rate as constant or linear without considering the variation across years is problematic. Similarly, the application of national or regional growth rate, crude birth rate or proportions to a subnational unit to estimate a target population regardless of local events or contexts will result in inaccurate denominator estimates.

The emergence of methods that rely on survey and high coverage indicators to estimate denominators for other RMNCH indicators is promising but also comes with notable limitations. These methods do not directly rely on population census projections and in settings where there is high coverage and reliable data, these methods can produce highly reliable and improved denominators [5]. However, in situations where there is low coverage or poor data quality of the related indicators, this method may not work. Completeness for this approach is defined as submission of report for a given month with less emphasis on the quality of the submitted report. The adjustment factor used by these methods is arbitrarily determined. The use of regional level coverage from surveys to estimate denominators for coverage at the district level does not account for the coverage inequalities across districts.

Denominator estimation for curative-based indicators rely on, and is limited to, individuals who use health facilities. If such groups are systematically different from people who do not access services from health facilities, the estimated coverage will not reflect the true coverage of the intervention in the population of interest.

The available methods are promising but have several limitations. There is the need for the exploration of alternative modern technology-based population estimation methods such as satellite imagery, geo-positioning and mobile phone call records to estimate the population size and migration at the subnational level[26, 55]. These methods are particularly useful in LMICs with large seasonal population migration and where traditional census-based projections may be inaccurate[26, 55]. Satellite imagery methods can be done remotely and can be used to validate or complement the existing methods to yield more accurate population estimates[26, 55] for RMNCH indicator denominators.

The division of a reported numerator by the census projected denominator without further adjustment could result in extremely high or low coverage estimates. Different methods were used to further adjust for extremely low or high RMNCH coverage indicators. Overall coverage adjustment was usually done when trying to estimate coverage at a high administrative level such as regions/provinces or national using coverage data from a lower level (district/county level). In some instances, the overall coverage was adjusted without necessarily determining whether the coverage issue relates to an inaccurately estimated numerator or denominator. It is important for future studies to delineate numerator related quality issues from denominator related quality challenges. The data source for numerators is different from denominators and the solution to address quality issues will be different between the two data points.

The annealing method, which uses weighted coverage from administrative and survey data to estimate coverage, is appealing because it produces standard errors making it possible to develop confidence bounds around the final estimated RMNCH indicator coverage. This method, according to the authors, produces an estimate that is more accurate and reliable compared to estimates from either data source alone. However, the authors did not indicate specifically which component of routine data challenge the method addresses or how to adjust for denominator and numerator issues in routine data. The authors did not state how missing data, over and under reporting and incomplete reporting in administrative data should be handled prior to applying the method.

This review has some limitations that should be noted. Titles and abstracts that were not published in English or indexed in PubMed or Scopus reviews were not captured. To the best of our knowledge, this is the first systematic review to examine the analytical methods that have been used to improve estimates of RMNCH intervention coverage using routine data from LMICs. This review provides a comprehensive overview of all the available analytical methods, their strengths and limitations which can be used by health departments across LMICs. This review also draws attention to the need to improve and develop new analytical methods that can increase the usage of routine data to inform local policies and RMNCH implementation plans.

## Conclusions

This paper summarizes the literature on the available analytical methods that can be used to estimate RMNCH intervention coverage in LMICs using routine data exclusively or together with survey data. The findings from this review show some advances in RMNCH coverage estimation methods using HMIS data. However, there are remaining gaps, particularly at the analytical stage, to accurately estimate the needed numerators, denominators, and coverage of RMNCH indicators using routine data. As countries continue to strive to close the gap and inequality in RMNCH coverage and meet SDG Goal 3, the consistent use of rigorous analytical methods that allow for the use of HMIS data to estimate RMNCH coverage at national and subnational levels is urgently needed. Improvement in routine data quality and analytical methods, together with advances in electronic information systems (data collection, reporting and transmission) and satellite imaging in LMICs will make HMIS data increasingly useful and efficient for the timely estimation of RMNCH intervention coverage at the national and subnational level. This will enable governments to identify gaps and develop policies and interventions to address such gaps in RMNCH indictor coverage. Even though the available methods are far from perfect, they pave the way for more research in this field.

## Data Availability

Not applicable.

## Abbreviations

ANC: Antenatal care
ART: Anti-retroviral therapy
BCG: Bacillus Calmette-Guerin
CRVS: Civil Registration and Vital Statistics
DTP: Diphtheria, Tetanus, Pertussis
HIV: HIV:Human Immuno-deficiency Virus
HMIS: Health Management Information System
LMICs: Low and Middle Income Countries
MDGs: Millennium Development Goals
OPV: Oral Polio Vaccine
RMNCH: Reproductive Maternal Neonatal and Child Health
WHO: World Health Organization
